# Physiological and Neuromuscular Fatigue after 3-Minute Lateral Shuffle Movement at Different Speeds and Distances

**DOI:** 10.5114/jhk/190145

**Published:** 2024-12-06

**Authors:** Mengde Lyu, Mingyue Yin, Ling Ding, Renhuan Tang, Zhili Chen, Shengji Deng, Yuming Zhong, Zhan Li, George P. Nassis, Yongming Li

**Affiliations:** 1 School of Athletic Performance, Shanghai University of Sport, Shanghai, China.; 2 School of Physical Education, Shanghai University of Sport, Shanghai, China.; 3 School of Human Sciences, University of Western Australia, Perth, Australia.; 4 College of Sport Science, University of Kalba, Kalba, Sharjah, United Arab Emirates.; 5 China Institute of Sport Science, Beijing, China.

**Keywords:** team sport, change of direction, neuromuscular fatigue

## Abstract

This study aimed to 1) investigate and compare physiological and neuromuscular fatigue after a 3-min lateral shuffle movement (LSM) at different speeds and distances, and 2) examine the relationship between the number of changes of direction (CODs) during LSM and fatigue-related variables. Twenty male college athletes (age: 20.9 ± 1.7 yrs, body mass: 72.8 ± 8.6 kg, body height: 177.9 ± 5.6 cm; mean ± SD) performed six LSM protocols at two speeds (1.8 and 2.0 m/s) and three distances (2.5, 5 and 10 m) in random order and on separate days. The heart rate (HR), blood lactate (BLa) concentration, the rating of perceived exertion (RPE) and the countermovement jump (CMJ) were assessed and recorded before and immediately after exercise. Neuromuscular fatigue was assessed with the decline in CMJ performance (CMJ_decline_). Results showed no interaction effect (speed*distance) for all variables. The distance was a significant factor influencing the HR (F = 12.663, p = 0.000, η_p_^2^ = 0.25), BLa concentration (F = 15.357, p = 0.000, η_p_^2^ = 0.288), CMJ_decline_ (F = 19.496, p = 0.000, η_p_^2^ = 0.339), and the RPE (F = 20.149, p = 0.000, η_p_^2^ = 0.347). Speed was also a significant factor influencing the HR (F = 6.367, p = 0.016, η_p_^2^ = 0.144), BLa concentration (F = 10.292, p = 0.000, η_p_^2^ = 0.213), CMJ_decline_ (F = 9.014, p = 0.005, η_p_^2^ = 0.192), and the RPE (F = 9.539, p = 0.004, η_p_^2^ = 0.201). CODs displayed moderate correlations with BLa concentration (r = 0.331, p < 0.001), CMJ_decline_ (r = –0.415, p < 0.001), and the RPE (r = 0.318, p < 0.001). These results suggest that physiological and neuromuscular fatigue would be greater with higher speed and shorter distance of LSM.

## Introduction

The lateral movements (LMs) encompass lateral shuffle, changes of direction (COD), and cutting, which is a prevalent kinetic pattern necessitating athletes to execute frequent shuffling maneuvers (Maurus et al., 2019). These movements enable athletes to traverse short distances laterally, alter their movement trajectory, and engage in high-intensity start-stop actions (Maurus et al., 2019; Subramanium et al., 2021; Luberecka 2022; Krzysztofik et al., 2023). A comprehensive study demonstrated that basketball athletes engage in LMs for approximately 18.1–42.1% of the total game duration, averaging between 0.62 and 1.42 s per high-intensity LMs and 0.84–1.98 s per low-intensity LMs (Taylor et al., 2017). During a basketball game, players execute more than 300 LMs and in each high-intensity LM they may cover distances ranging between 2.28 and 4.24 m (Scanlan et al., 2011; Taylor et al., 2017).

Previous research suggested that the intensity of LM was dependent on the frequency of 180°-COD during its process (Taylor et al., 2017). This also implied that LM is a type of movement that relies on the continuous stretch-shortening cycle (SSC) capability of the lower limbs (Nicol et al., 2006). Prior investigations have illuminated the prevalence of COD, accelerations, and decelerations in team and racket sports competitions, wherein SSC movements have been identified as potential catalysts for substantial fatigue, and at the same time, fatigue may affect the efficiency of the SSC by reducing neural drive, decreasing muscle fiber contraction rates, and weakening force output (Komi, 2000). This highlights the interaction between the SSC and fatigue and implies the negative impact of fatigue on athletic performance.

Notably, the different fatigue induced by COD has predominantly been explored in the context of forward and backward movements, with limited attention directed towards comprehending these responses within the realm of LM. Hader et al. (2014) observed an increase in blood lactate (BLa) and the rating of perceived exertion (RPE), and a decline in the height of countermovement (CMJ) and a drop jump in athletes when introducing COD to straight running (Hader et al., 2014). Similarly, Dellal et al. (2010) integrated straight-line running with various bouts of COD and reported elevated physiological and perceptual demands (Dellal et al., 2010). However, in the study of Gao et al. (2022), where the physiological and perceptual responses to forward, forward-backward, and LM in a 5-m shuttle running at a speed of 6 km•h^−1^ were examined, they found that BLa and the RPE of LM were higher than of forward and forward-backward shuttle running (Gao et al., 2022).

Since speed and distance of LMs in actual games are uncertain, as well as the number of COD during LMs, these uncertainties can lead to fatigue in various aspects. Therefore, it is challenging for coaches to design targeted training for LMs in daily routines. For instance, when coaches aim to enhance physiological and neuromuscular capacities of athletes through LMs which closely mimic the demands of actual gameplay, they should consider how to structure training sessions and whether altering the distance, speed, and CODs during LM training can effectively target specific aspects of athletic performance. Given the significance of LMs in various sports, there is currently a lack of systematic research on physiological and neuromuscular fatigue caused by LMs.

Therefore, we designed a specific lateral shuffle movement (LSM) task at different speeds and distances, and the aim of this study was: (1) to assess physiological (heart rate, BLa, and RPE) and neuromuscular (CMJ) fatigue following LSM at different speeds and distances for the same movement duration, and (2) to investigate the relationship between COD and fatigue-related variables. Based on the rationale, we hypothesized that: (1) LSM would significantly induce both physiological and neuromuscular fatigue with the increase in distance and speed conditions; and (2) there would be a correlation between COD and fatigue-related variables.

## Methods

### Participants

Twenty young male college athletes (age: 20.9 ± 1.7 yrs, body mass: 72.8 ± 8.6 kg, body height: 177.9 ± 5.6 cm; mean ± SD), who were basketball, soccer, tennis, and badminton players volunteered to participate in this study. The inclusion criteria were as follows: (1) consistent engagement in regular training sessions at least three times per week over the preceding four weeks; (2) free of lower limb injury for the last six months and no history of previous lower limb surgery; (3) willingness to undergo the experimental tests and provide signed informed consent. Prior to formal testing, all participants signed the informed consent. This study was conducted following the principles of the Declaration of Helsinki, and approved by the Institutional Ethics Committee of the Shanghai University of Sport (protocol code: 102772023RT104; approval date: 13 October 2023).

### Design

Each participant executed 6 LSM protocols in random order, apart. Each LSM protocol lasted for 3 min (Kramer et al., 2019) and varied in running speed (1.8 m/s and 2.0 m/s) (Scanlan et al., 2011) and in distance covered (2.5 m, 5 m and 10 m). The running protocols were: 2.5-m LSM at 2.0 m/s, 2.5-m LSM at 1.8 m/s, 5-m LSM at 2.0 m/s, 5-m LSM at 1.8 m/s, 10-m LSM at 2.0 m/s, and 10-m LM at 1.8 m/s. Testing took place on an indoor running track, under stable environmental conditions (average temperature of 21.6 ± 1.2°C, range: 19–23°C; average humidity of 61.8 ± 7.5%, range: 55–72%).

Physiological fatigue variables included the heart rate (HR), BLa and the RPE. Neuromuscular fatigue (NMF) was quantified using the average height of the CMJ. A uniform 10-min warm-up routine, comprising jogging, dynamic stretching, nd three submaximal CMJs, was administered prior to the commencement of the LSM tests. The HR was continuously monitored throughout the entire duration of LSM. BLa, CMJ, and RPE assessments were conducted both pre- and post-LSM program.

### Measurements

**Figure 1:**
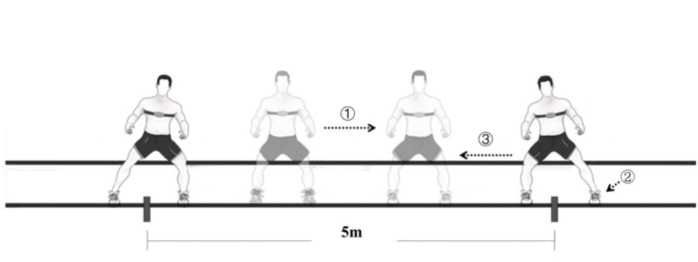
The step of continuous 3-min LSM (e.g., 5-m LSM). *① shuffle; ② cutting; ③ 180° change of direction*. Abbreviation: LSM, lateral shuffle movement

LSM encompassed three distinct distances and two speeds, as outlined in Table 1. During the execution of LSM, participants regulated their movement speed guided by audio cues, shuttling horizontally between two demarcated lines. The auditory signal prompted participants to position their body center in alignment with one of the two lines at each cue (Figure 1). The prescribed action was maintained at a stance where both feet were parallel and shoulder-width apart, with the knees flexed at approximately 60° (Wu et al., 2022).Importantly, throughout the exercise, the tester provided consistent reminders to participants, emphasizing the necessity to sustain the specified knee joint angle.

**Table 1:** CODs and the total moving distance of each LSM.

	2.5 m		5 m		10 m	
	1.8 m/s	2.0 m/s	1.8 m/s	2.0 m/s	1.8 m/s	2.0 m/s
CODs (number)	128	144	64	72	32	36
Total moving distance (m)	324	360	324	360	324	360

Abbreviation: LSM, lateral shuffle movement; CODs, number of changes of direction

The constraints imposed by the specified pace and duration for each distance in LSM protocols yielded distinct characteristics (Table 1). Longer distances in LSM protocols manifested a greater frequency of shuffles and a reduced occurrence of shuttles. For instance, the number of CODs in the 2.5-m LSM protocol was twice compared to that of the 5-m LSM protocol when the speed remained constant. Additionally, higher speeds resulted in a higher number of shuttles, which is indicative of a greater frequency of CODs.

Capillary blood samples were obtained from the participant's earlobe both before and immediately after the post-CMJ test, with subsequent analysis of BLa conducted using the EKF lactate analyzer (Biosen S_line, EKF Diagnostic, Magdeburg, Germany). The HR was continuously monitored throughout all LSM protocols utilizing the Polar H9 device (Polar Electro, Kempele, Finland). The RPE was evaluated before and immediately after LSM protocols using the Borg's 6–20 scale (Borg, 1998).

The difference in average height of CMJs (CMJ_decline_) before and after exercise was used to evaluate neuromuscular fatigue, and it has been widely used in monitoring neuromuscular status (Claudino et al., 2017). The CMJ was measured using a phone app (MY JUMP2) which has been proven as a reliable tool to measure vertical jump performance [*r* = 0.99; ICC = 0.99] (Balsalobre-Fernández et al., 2015; Sahin-Uysal et al., 2023). Following a standardized warm-up, participants performed 3 practice submaximal CMJs and 3 maximal CMJs before and immediately after the LSM protocols. The pre- and post-CMJ was separated by 30 s. The average height was used in the final analysis (Claudino et al., 2017).

### Statistical Analysis

Descriptive data were expressed as mean ± standard deviation (SD). All data were tested for normal distribution and homogeneity of variance using the Shapiro-Wilk test and the Levene's method, respectively. A two-way repeated measures ANOVA (speed × distance) was employed to check the main effects and interactions for the HR, BLa, CMJ_decline_, and the RPE. Subsequently, Tukey post hoc tests were conducted to locate the differences between LSM protocols when significant effects were identified. Partial Eta Squared (*η_p_*^2^) was calculated as the effect size index and classified as small (0.1 < *η_p_*^2^ < 0.06), moderate (0.06 ≤ *η_p_*^2^ < 0.14), or large (*η_p_*^2^ ≥ 0.14). The relationships between the HR, BLa, CMJ_decline_, the RPE and CODs were analyzed using the Pearson correlation analysis. The magnitude of the correlation between test variables was determined as: ≤ 0.1 (trivial); 0.1–0.3 (small); 0.3–0.5 (moderate); 0.5–0.7 (large); 0.7–0.9 (very large) and 0.9–1.0 (almost perfect) (Hopkins et al., 2009).Statistical significance was set at *p* < 0.05. The statistical analyses were conducted using IBM SPSS software version 26.0 (IBM, Armonk, New York, USA).

## Results

**Figure 2:**
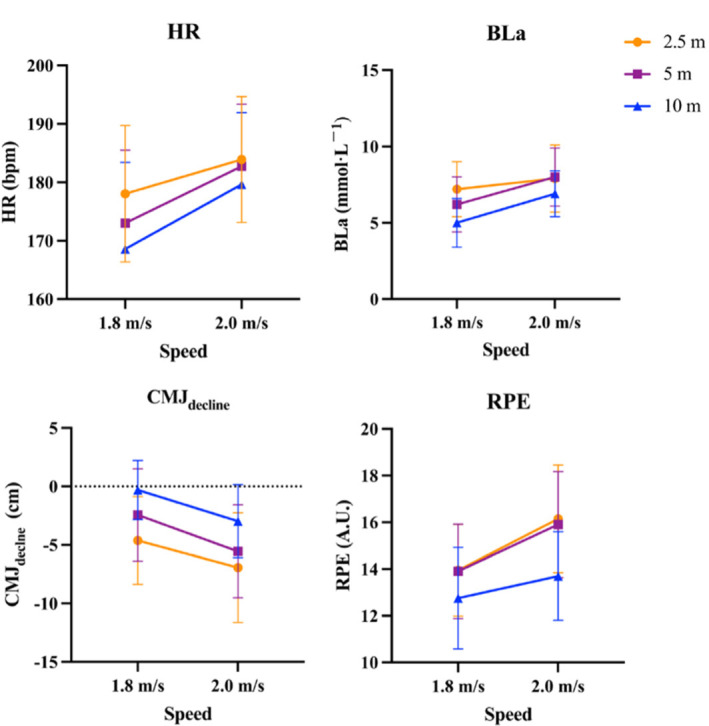
Physiological and neuromuscular fatigue of LSM at different speeds and distance (Mean ± SE). Abbreviation: LSM, lateral shuffle movement; HR, heart rate (bpm); BLa, blood lactate concentration (mmol•L^−1^); CMJ_decline_, the decline in average height of the countermovement jump (cm); RPE, rating of perceived exertion (A.U.)

The distance of the LSM had a significant effect on the HR (*F* = 12.663, *p* = 0.000, *η_p_*^2^ = 0.25), BLa (*F* = 15.357, *p* = 0.000, *η_p_*^2^ = 0.288), CMJ_decline_ (*F* = 19.496, *p* = 0.000, *η_p_*^2^ = 0.339), and the RPE (*F* = 20.149, *p* = 0.000, *η_p_*^2^ = 0.347), indicating a notable impact of the independent variable on dependent variables. The higher was the distance of each shuttle, the lower was the physiological and neuromuscular fatigue (Figure 2). Additionally, speed also emerged as a significant factor influencing the HR (*F* = 6.367, *p* = 0.016, *η_p_*^2^ = 0.144), BLa (*F* = 10.292, *p* = 0.000, *η_p_*^2^ = 0.213), CMJ_decline_ (*F* = 9.014, *p* = 0.005, *η_p_*^2^ = 0.192), and the RPE (*F* = 9.539, *p* = 0.004, *η_p_*^2^ = 0.201). It can be clearly seen that the main effects of the distance factor on the HR, BLa, CMJ_decline_, and the RPE were more pronounced compared to the main effects of the speed factor. No interaction effect (speed*distance) was found for all variables.

**Figure 3:**
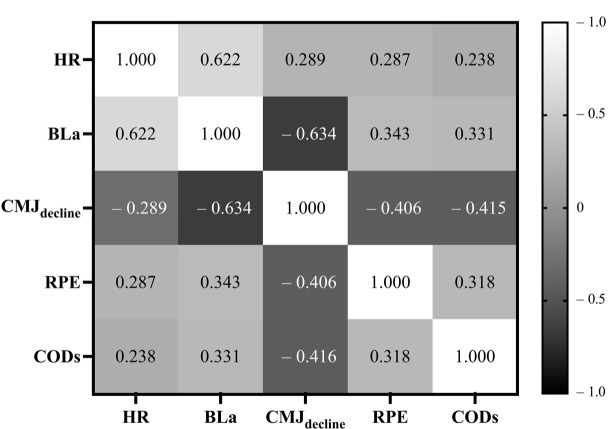
The correlation analysis between the HR, Bla, CMJ_decline_, te RPE and CODs. Abbreviation: HR, heart rate (bpm); BLa, blood lactate concentration (mmol•L^−1^); CMJ_decline_, the decline in average height of the countermovement jump (cm); RPE, rating of perceived exertion (A.U.); CODs, the number of changes of direction (A.U.)

The Pearson correlation analysis revealed significant relationships among the HR, BLa, CMJ_decline_, the RPE, and CODs, with all correlations exhibiting statistical significance (all *p* < 0.01). Notably, BLa exhibited a substantial correlation with both CMJ_decline_ (*r* = –0.634, *p* < 0.001) and the HR (*r* = 0.622, *p* < 0.001). Furthermore, CODs displayed moderate correlations with BLa (*r* = 0.331, *p* < 0.001), CMJ_decline_ (*r* = –0.415, *p* <0.001), and the RPE (*r* = 0.318, *p* < 0.001). These findings underscored the intricate interplay and interdependence among physiological and neuromuscular variables, as well as their relationship with the frequency of CODs (Figure 3).

## Discussion

The main findings of the present study are that: 1) as the speed of LSM increased, physiological and neuromuscular fatigue became more severe; 2) as the distance of LSM decreased, physiological and neuromuscular fatigue also became more severe; 3) the HR, BLa, CMJ_decline,_ the RPE and CODs exhibited significant correlations with each other.

Based on our findings, alterations in both speed and distance influenced physiological and neuromuscular fatigue, demonstrating comparable trends. The increase in speed led to greater effort exerted by participants to accelerate and decelerate in order to maintain movement, while the decrease in distance caused an increase in the number of COD actions throughout the LSM protocols. Both these two factors contributed to more pronounced fatigue. Even though statistical results showed that the distance factor was more significant than the speed factor, we cannot directly conclude that the distance factor is more influential. Due to the different proportions of increases in speed and distance, with speed exhibiting a much lower percentage increase compared to distance (11.1% vs. 100%), significant main effects were still observed in our results. Therefore, we believe that increasing the speed of LSM is likely to be more effective in inducing fatigue.

The correlation analysis indicated significant relationships between CODs and the HR, BLa, the CMJ, and the RPE (all *p* < 0.01), demonstrating that increases in CODs due to distance reduction in LSM lead to a higher degree of fatigue. This is consistent with previous research findings regarding the association between COD and fatigue. For example, Ashton and Twist (2015) found that an increased number of repetitions during prolonged intermittent shuttle running in female basketball players correlated with elevated mean HR, BLa, and the RPE. Hader et al. (2014) observed a commensurate augmentation in both physiological and neuromuscular responses among athletes when introducing COD to straight running. Similarly, Dellal et al. (2010) integrated straight-line running into COD maneuvers, revealing heightened physiological and perceptual demands in shuttle running scenarios. The correlation between CODs and the CMJ was the highest (*r* = –0.416, *p* < 0.01). This implies that the increased frequency of CODs led to more pronounced NMF, which is consistent with the findings of Taylor et al. (2017), who suggested that the intensity of LSM was influenced by the quantity of CODs executed. Research indicates that COD movements relying on the SSC could lead to significant NMF due to the rapid alternation of muscle contractions between concentric and eccentric phases (Nicol et al., 2006). Moreover, studies suggest that there is an interaction between the SSC and NMF, which means that as fatigue increases, the SSC ability of the muscles may decrease (Komi, 2000; Wadden et al., 2012). This is because fatigue can affect the efficiency of the SSC by reducing neural drive, decreasing muscle fiber contraction rates, and weakening force output (Komi, 2000). Additionally, we found a significant correlation between CMJ_decline_ and Bla (*r* = –0.634, *p* < 0.01). Previous findings have also demonstrated significant correlations between CMJ_decline_ and Bla in various athletic populations including sprinters (Jimenez-Reyes et al., 2016) and soccer players who underwent repeated sprint ability testing (Morcillo et al., 2015). Our results once again underscore the effectiveness of the decline in vertical jump height as a valid indicator of fatigue.

Incorporating more CODs or faster speeds into LSM training will induce physiological and neuromuscular fatigue. This consideration can be strategically integrated into the design of training programs by coaches. By adding heightened complexity in movement patterns, athletes are exposed to the challenges of fatigue (Barrera-Dominguez et al., 2024). This, in turn, contributes to an overall improvement in their athletic capabilities. Coaches can increase the stimulation to athletes' bodies to adapt to the demands of competition by reducing the distance of continuous LM and increasing the number of CODs within it. However, when the CODs reach a certain time, inducing fatigue through increased LM speed can be used to avoid more CODs, thus reducing the neuromuscular load and fatigue on athletes, thereby preventing sports injuries. Furthermore, these research findings carry noteworthy implications for the analysis of external and internal training loads among practitioners within team settings. For instance, when evaluating exercises covering the same distance (external load), the inclusion of LM drills will cause elevated physiological and neuromuscular fatigue. This inference suggests that LMs introduce higher internal effort. Consequently, these results can be instrumental in refining and optimizing team training regimens, ensuring that athletes achieve optimal adaptations to meet diverse athletic demands.

Our study has certain limitations which need to be acknowledged. Firstly, participants were from different sports disciplines, and only male athletes were included. The overall heterogeneity and gender differences need to be considered. This heterogeneity introduces the potential for confounding factors playing a role in the final results, given that the demands and protocols of LSM may significantly differ across various sports. Secondly, despite instructing participants to control their center of body weight with the knees flexed at approximately 60°, we could only provide verbal reminders during testing. It remains challenging to ascertain whether all participants consistently adhered to the prescribed movement.

## Conclusions

Our research findings indicate that physiological and neuromuscular fatigue would be more pronounced with higher speed and shorter distance of LSM. The CMJ_decline_ was significantly correlated with CODs and BLa. Based on these findings, coaches can better design training programs and monitor players’ fatigue.
